# Association between *p*53 Codon 72 (Arg72Pro) Polymorphism and Primary Open-Angle Glaucoma in Iranian Patients

**DOI:** 10.6091/ibj.1379.2014

**Published:** 2015-01

**Authors:** Hossein Neamatzadeh, Reza Soleimanizad, Masoud Zare-Shehneh, Saba Gharibi, Abolfazl Shekari, Amir Bahman Rahimzadeh

**Affiliations:** 1 *Hematology, Oncology and Genetic Research Center, Shahid Sadoughi University of Medical Sciences and Health Services, Yazd, Iran;*; 2 *Dept. of Ophthalmology, Shahid Sadoughi University of Medical Sciences and Health Services, Yazd; Iran; *; 3 *Dept. of Medical Genetics, Shahid Sadoughi University of Medical Sciences and Health Services, Yazd, Iran; *; 4 *Dept. of Medical Genetics, Zanjan University of Medical Sciences, Zanjan, Iran;*; 5 *Dept. of Hematology, Tabriz University of Medical Sciences, Tabriz, Iran*

**Keywords:** Primary open-angle glaucoma (POAG), Glaucoma, p53, Codon 72, Iran

## Abstract

**Background: **Glaucomatous neuropathy is a type of cell death due to apoptosis. The *p53* gene is one of the regulatory genes of apoptosis. Recently, the association between the *p53* gene encoding for proline at codon 72 and primary open-angle glaucoma (POAG) has been studied in some ethnic groups. This study is the first association analysis of POAG and *p53* codon 72 polymorphism in Iranian patients. **Methods: **A cohort of 65 unrelated patients with POAG (age range from 12-62 years, mean ± SD of 40.16 ± 17.51 years) and 65 unrelated control subjects (without glaucoma, age range of 14-63 years, mean ± SD of 35.64 ± 13.61 years) were selected. In Iranian POAG patients and normal healthy controls, the *p53* codon 72 polymorphism in exon 4 was amplified using polymerase chain reaction. The amplified DNA fragments were digested with the BstUI restriction enzyme, and the digestion patterns were used to identify the alleles for the polymorphic site. **Results: **Comparisons revealed significant differences in allele and genotype frequencies of Pro72Arg between POAG patients and control group. A higher risk of POAG was associated with allele Pro (OR = 2.1, 95% CI = 1.2–3.4) and genotype Pro/Pro (OR = 3.9, 95% CI = 0.13-12.7). **Conclusion: **The *p53* Pro72 allele was more frequent in Iranian POAG patients than in the control group (*P*<0.05). The present findings show that the individuals with the Pro/Pro genotype may be more likely to develop POAG. However, additional studies are necessary to confirm this association.

## INTRODUCTION

Glaucoma is currently the main cause of irreversible, chronic, degenerative optic neuro-pathy, which affects approximately 70-80 million people worldwide [1, 2]. This disease is the second leading cause of vision loss, and the number of people suffer from this disease is expected to increase due to the aging [3]. Indeed, glaucoma comprises a group of neurodegenerative disorders that involve apoptotic death of retinal ganglion cells. 

Glaucoma can be roughly divided into three main types: open-angle, closed-angle, and developmental [4]. Primary open-angle glaucoma (POAG) is the most common type of glaucoma characterized by a complex inheritance, slow, and irreversible apoptotic death of retinal ganglion cells, a unique optic nerve neuropathy resulting in loss of vision, adult onset, a gonio-scopically open-angle, and a reduced outflow facility that originates elevated intraocular pressure [3, 5, 6]. 

It is known that POAG is a multifactorial disease in which genetic and environmental factors are involved [7]. Various risk factors have identified for develop-ment of open-angle glaucoma, including age, elevated intraocular pressure, exfoliation syndrome, race, myopia, diabetes, and decreased perfusion pressure [8, 9]. However, a positive family history remains among the most important ones established for POAG. Genetic studies have identified that specific genes (such as *MYOC*, *ASB10*, *WDR36*, *NTF4*, and *TBK1*) contribute to the pathogenesis of POAG [10-12].

The common polymorphism of *p53* at codon 72, either encoding proline or arginine, has known as a genetic factor associated with clinical outcome or *several different* neoplasms in humans, *such* as *lung cancer*, colorectal *cancer*, *thyroid cancer*, nasopharyn-geal *cancer*, and oral one [13]. Studies have shown that *p53* gene polymorphisms may involve in POAG pathogenesis [7, 11]. The individual role of *p53* codon 72 polymorphism in POAG has also been investigated in POAG pathogenesis [10]. For this polymorphism, the G allele encodes an arginine at position 72 of the protein (where there is normally a proline). The SNP is commonly called the Arg72 allele, although P72R and Arg72Pro are also common in the literature. For the first time in 2002, Lin *et al. *[14] reported that the *p53* codon 72 polymorphism is associated with POAG. However, the association between this polymorphism and POAG remains controversial. Fan *et al. *[12] have suggested that variants in *p53* are risk factors for POAG, whereas variants in other studied genes are not major risk factors for POAG, at least in Chinese population. It has been also indicated that this variation in association could be due to different factors, such as ethnicity, sample size, poorly characterized controls, and clinical heterogeneity between different populations [15]. Therefore, more studies are necessary to gain further insight into the relationship between *p53* codon 72 polymorphism and POAG. 

This investigation analyzes, for the first time, the relationship between *p53* codon 72 polymorphism and POAG in Iranian POAG patients. Therefore, this study attempts to investigate whether codon 72 polymorphisms in *p53* were associated with POAG in Iranian population.

## MATERIALS AND METHODS

***Patients. ***All of the participating subjects were recruited from glaucoma outpatients between December 2011 and October 2013. They were selected from three different referral centers in Yazd, Tehran, and Isfahan (Iran). A cohort of 65 unrelated patients without familial background with POAG, ranging from 12-62 years of age (mean ± SD of 40.16 ± 17.51 years), and 65 unrelated normal controls were over matched population with a similar age range of 14-63 years (mean ± SD of 35.64 ± 13.61 years) who had no personal or family history of any cancer to date. All controls underwent a complete ophthalmic examination in order to exclude individuals with glaucoma from the control group. Then, they were confirmed to have no visual complaints and intraocular pressure of <22 mmHg with a normal desk appearance. Control individuals with a family history of glaucoma were excluded. 

For the purposes of this study, the narrow definition of affected POAG status was based on open angles, glaucomatous optic neuropathy, and visual field defects consistent with glaucoma. Glaucomatous optic neuropathy was defined as a narrowed neuroretinal rim, notching of the neuroretinal rim, and/or marked asymmetry in the cup-to-disc ratio. Glaucomatous visual field defects were based on the Glaucoma Hemifield Test and clinician interpretation. The diagnosis criteria used for patient recruitment were increase in intraocular pressure and glaucomatous damage to the optic nerve head, and/or glaucomatous damage of the visual field. According to the exfoliative glaucoma patients, the additional criterion of presence of pseudo exfoliative material on e.g. the iris or lens was required for diagnosis. Exclusion criteria included eye surgery and use of glaucoma eye drops for more than two weeks. All information was devoid of identifiers and kept in a database. The data collection was in accord with an approval by the Shahid Sadoughi Medical Science University (Yazd, Iran). 

***The p53 codon 72 polymorphism genotyping. ***Venous blood (20 ml) was extracted from all participants and distributed into 4 ml EDTA tubes and stored at -30^○^C. Genomic DNA was prepared using the Qiagen kit (Tehran, Iran). The status of the *p53* Arg72Pro was determined by using BstUI RFLP analysis. First, a 296-bp fragment was amplified using forward (5'-TTGCCGTCCCAAGCA ATGGATGA-3') and reverse (5'-TCTGGGAAGGGACAGAAGATG AC-3') primers. The reaction mixture (50 μl) contained 0.1 μg genomic DNA, 1 U Taq DNA polymerase, 10 pmol of each primer, 200 Μm of each dNTP, and 1.5 mM MgCl_2_. The amplification program was comprised of 5 min of denaturing at 95ºC, followed by 30 cycles of 30 s at 95ºC, 30 s at 57ºC, and 1 min at 72ºC with a final step at 72ºC for 7 min. The resulting 312 bp PCR products of *p53* exon 4 were digested with BstUI according to the manufacturer’s protocol. Briefly, 10 μl PCR product was mixed with 10 U BstUI restriction enzyme (Fermentas, Lithuania) in 1× buffer, containing 10 mM Tris, 10 mM MgCl_2_, 100 mM KCl, and 0.1 mg/ml BSA, pH 8.5. After being separated in 4% agarose gel electrophoresis containing ethidium bromide, digestion products were visualized under UV transilluminator. The codon 72 wild-type Arg/Arg homozygote product is cleaved by this endonuclease and yields one 259-bp and one 53-bp band. In addition, the Pro/Pro homozygote is not cleaved by the enzyme and yields a single 312-bp band, while the Arg/Pro heterozygote contains all three bands.

***Statistical Analysis. ***Statistical analysis was performed using statistical software package SPSS 17.0 software. The Hardy-Weinberg equilibrium was estimated separately for patients and controls. For statistical analysis, the distribution of this polymer-phism in the control and POAG groups was compared using the chi-square test. The association between *p53* codon 72 polymorphism and POAG was assessed by computing the odds ratio (OR) and 95% confidence intervals (95% CI). The results were considered statistically significant when the probability of findings occurring by chance was less than 0.05 (*P*<0.05).

**Table 1:** Distribution of *p53* Arg72Pro polymorphism in POAG patients and controls

***p53*** ** Arg72Pro**	**POAG (%)**	**Controls (%)**	**OR** ^a^ ** (95% CI)**	***P*** ** value**
**Genotypes** Arg/Arg Arg/Pro Pro/Pro	17(26.1%) 27(41.6%) 21(32.3%)	25(38.5%) 32(49.2%) 8(12.3%)	1 2.2 (0.89-5.1) 3.9 (0.13-12.7)^*^	0.008
				
**Allele** Arg Pro	61(0.47%) 69(0.53%)	82(0.63%) 48(0.37%)	1.00 2.1(1.2–3.4^)*^	- -

POAG, primary open-angle glaucoma; OR^a^, odds ratio; 95%CI, 95% confidence intervals (*P<0.05).

## RESULTS

**Fig. 1:**
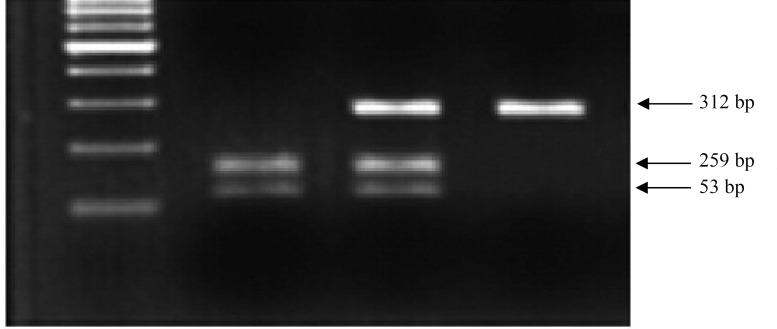
Genotype analysis by digestion of RFLP-PCR products. Lane 1 is Pro homozygote sample, lane 2 is Arg/Pro heterozygote, and lane 3 is homozygote samples for Arg allele. Lane M, 100 bp DNA ladder (Fermentas).

The frequencies of the genotypes in the control group and the POAG patients are shown in Table 1. The proline allele is identified by the presence of a single fragment of 312 bp, and the arginine allele by two fragments of 259 and 53 bp, respectively. Hetero-zygous samples displayed all three fragments (Fig. 1). Using the chi-square test, the distribution of the *p53* codon 72 polymorphism was compared, and a significant difference was found between groups POAG patients and controls (*P*<0.008). The distribution of the genotypes in the POAG group revealed 17 (26.1%) Arg homozygotes, 27 (41.6%) Arg/Pro heterozygotes, and 21 (32.3%) Pro homo-zygotes. The distribution of the genotypes in the control group revealed 25 (38.5%) Arg homozygotes, 32 (49.2%) Arg/Pro heterozygotes, and 8 (12.3%) Pro homozygotes. 

The allelic frequencies in the POAG group were 61 (0.47%) Arg and 69 (0.53%) Pro, while in the control group were 82 (0.63%) Arg and 48 (0.37%) Pro. The frequency of the Pro allele was significantly higher in the POAG group. In this study, there was a significant association between the Pro allele of *p53* Arg72Pro and POAG in Iranian patients. A higher risk of POAG was associated with allele Pro (OR = 2.1, CI 1.2–3.4) and genotype Pro/Pro (OR = 3.9, CI 0.13-12.7). 

## DISCUSSION

The ultimate goal for glaucoma genetic research is to identify the specific set of gene mutations that confer high-risk of developing glaucoma, which could then be used to screen patients before clinical phenotypes manifestation. It is suggested that POAG is most likely caused by the interactions of multiple genes and environmental factors. It appears that most of the association studies for POAG have investigated only single genes or single gene alleles without accounting for contributions from gene-gene and gene-environment interactions [16].

The pathogenesis of POAG is genetically hetero-geneous and complex. Although, it has been shown that several genetic loci and genes have associated with POAG, the major genes that confer significant susceptibility remain unknown [7]. Interestingly, *p53* has been involved within the development of POAG [7]. In recent decade, *p53* as a main candidate susceptibility gene to the glaucoma has studied broadly [17]. There have been inconsistent reports regarding the increased risk of glaucoma and genetic variations within *p53* [14]. Originally, an association was detected between POAG and SNP in exon 4 of *p53* at codon 72 in a Chinese population [14]. However, two other studies conducted in Australia and India did not report such association in POAG patients [17, 18].

The *p53* ability to trigger apoptosis depends on the residue occupying position 72 in the polypeptide chain. The *p53* isoform with Arg72 more efficiently induces apoptosis, and its content in the mitochondrial fraction is almost one order of magnitude higher than the content of *p53* with Pro72 [19]. In addition, the Arg72 isoform of *p53* more efficiently activates and interacts with *p53* as compared to the Pro72 isoform. In contrast, the Pro72 allele appears to induce a higher level of G1 arrest than the Arg72 allele. Therefore, it seems that the Arg72 and Pro72 alleles are functionally distinct, and these differences may influence the risk of cancer development [20]. Dumont *et al. *[21] reported that the Arg72 allele had up to 15 fold increased apoptotic ability compared with the Pro72 allele in both inducible cell lines and cells with endogenous *p53* homozygous for each allele. Storey *et al. *[22] found that when *p53* was degraded by human papilloma virus, the Pro form was seven times longer than the Arg form. Therefore, the hypothesis is that the Arg form of *p53* in residue 72 may be responsible for the less potent effects when the cell must be replicated. Therefore, the results of this study indicated that the Pro form allele is a significant risk factor for POAG [22]. 

Epidemiological studies have reported that the Arg72 allele is more common in Northern Europeans than in Africans or African-Americans [23, 24]. The Pro/Pro genotype is found in 47% of sub-Saharan Africans and 28% of Japanese and 8% of European Whites, a population with an increased prevalence of normal tension glaucoma [12]. It is hypothesized that *p53* codon 72 alleles were latitude dependent. Interestingly, it has been shown that this latitude dependency is tightly associated with winter temperature [23, 24]. Furthermore, it seems that *p53* codon 72 polymorphism may be involved in the pathogenesis of POAG in Asians, but not in Caucasians [24]. These findings display the potential role of ethnic difference in genetic background and the environment where they live in.

This investigation is the first association study between the POAG and *p53* gene in the Iranian population. The results have shown that the distribution of the *p53* gene codon 72 polymorphism in Iranian POAG patients and the healthy control group is significantly different (*P* = 0.007) (Table 1), confirming the idea of the relation between apoptosis and neuropathy. As mentioned previously the associ-ation of the codon 72 polymorphism in *p53* gene has not been compatible in most studies. In a meta-analysis study by Guo *et al. *[25], it has been shown that *p53* codon 72 (Pro/Pro vs. Arg/Pro + Pro/Pro‏) and intron 316-bp insertion (Ins vs. Del) polymorphisms were associated with increased risk for POAG. However, no significant association was identified between rs1042522 and POAG in studies carried out in Indian, Australian, Japanese, Turkish, and Brazilian populations [16]. It is possible that the polymorphism is associated with the Iranian POAG patients, but not with other ethnic groups [18]. The results showed that the Pro allele was prevalent in Iranian POAG patients (OR = 2.389, 95% CI: 1.14 to 5.01) (Table 1). In contrast, Daugherty CL *et al. *[7] have found that the Arg allele is associated with increased risk of POAG. Some reports have revealed that the Pro allele homozygote is a risk factor for other conditions such as lung and hepatocellular carcinoma [7, 22]. Chen *et al*. [26] noted a significant association between Pro homozygotes and invasive bladder cancer in Chinese people. Furthermore, researchers have found that lung carcinoma patients with either *p53* Arg or Pro homozygotes have worse prognoses when compared with patients with the heterozygous form [26]. 

Population studies of the association between the *p53* Pro72Arg polymorphism with nerve diseases have yielded rather discrepant data. It has been reported that Pro and Arg alleles have association with neuropathy in POAG and early onset Leber’s hereditary optic neuropathy patients, respectively [26]. Moreover, the association between Pro72Arg with glaucoma has not been confirmed in different works [24]. This diversity may be due to racial variation. The studies mentioned above might suggest that the dominant *p53* Pro form is a risk factor for disease in the Chinese population. Nevertheless, it is suspected that the Pro form of *p53* gene codon 72 induces the instability of ocular ganglion cells, and the Pro form allele fails to protect ganglion cells from apoptosis. The Arg allele was observed to be related to cancer. It is suggested that the proteomics studies may find the exact effect of Pro on POAG [14].

In summary, the *p53* Arg72 allele was more frequent in the Iranian POAG patients compared with those in the control group (*P*<0.05). The result of this study suggests that patients with the Pro/Pro genotype may be more likely to develop POAG; however, additional studies are necessary to confirm this association.

## References

[1] Resnikoff S, Pascolini D, Etya'ale D, Kocur I, Pararajasegaram R, Pokharel GP (2004). Global data on visual impairment in the year 2002. Bull World Health Organ.

[2] Leske MC, Heijl A, Hussein M, Bengtsson B, Hyman L, Komaroff E (2003). Factors for glaucoma progression and the effect of treatment: the early manifest glaucoma trial. Arch Ophthalmol.

[3] Quigley HA, Broman AT (2006). The number of people with glaucoma worldwide in 2010 and 2020. Br J Ophthalmol.

[4] Shields M, Ritch R, Lowe RF, Ritch R, Shields MB, Krupin T (1996). Classifications and mechanisms of the glaucomas. The Glaucomas.

[5] Blanco-Marchite C, Sánchez-Sánchez F, López-Garrido MP, Iñigez-de-Onzoño M, López-Martínez F, López-Sánchez E (2011). WDR36 and P53 gene variants and susceptibility to primary open-angle glaucoma: analysis of gene-gene interactions. Invest Ophthalmol Vis Sci.

[6] Nickells RW (2007 Nov-Dec). Ganglion cell death in glaucoma: from mice to men. Vet Ophthalmol.

[7] Daugherty CL, Curtis H, Realini T, Charlton JF, Zareparsi S (2009). Primary open angle glaucoma in a Caucasian population is associated with the p53 codon 72 polymorphism. Mol Vis.

[8] Moore D, Harris A, Wudunn D, Kheradiya N, Siesky B (2008). Dysfunctional regulation of ocular blood flow: A risk factor for glaucoma?. Clin Ophthalmol.

[9] Kwon YH, Fingert JH, Kuehn MH, Alward WL (2009). Primary Open-Angle Glaucoma. N Engl J Med.

[10] Scheetz TE, Fingert JH, Wang K, Kuehn MH, Knudtson KL, Alward WL, Boldt HC (2013). A genome-wide association study for primary open angle glaucoma and macular degeneration reveals novel Loci. PLoS One.

[11] Wiggs JL (2007). Genetic etiologies of glaucoma. Arch Ophthalmol.

[12] Fan BJ, Wiggs JL (2010). Glaucoma: genes, phenotypes, and new directions for therapy. J Clin Invest.

[13] Dholariya S, Zubari M, Ray PC, Gandhi G, Khurana N, Yadav P (2013). TP53 gene polymorphism in epithelial ovarian carcinoma patients from North Indian population and its Pro/Pro variant is potentially contributing to cancer susceptibility. J Genet Syndr Gene Ther.

[14] Lin HJ, Chen WC, Tsai FJ, Tsai SW (2002). Distributions of p53 codon 72 polymorphism in primary open angle glaucoma. Br J Ophthalmol.

[15] Rao KN, Nagireddy S, Chakrabarti S (2011). Complex genetic mechanisms in glaucoma: an overview. Indian J Ophthalmol.

[16] Fan BJ, Liu K, Wang DY, Tham CC, Tam PO, Lam DS (2010). Association of polymorphisms of tumor necrosis factor and tumor protein p53 with primary open-angle glaucoma. Invest Ophthalmol Vis Sci.

[17] Dimasi DP, Hewitt AW, Green CM, Mackey DA, Craig JE (2005). Lack of association of p53 polymorphisms and haplotypes in high and normal tension open angle glaucoma. J Med Genet.

[18] Acharya M, Mitra S, Mukhopadhyay A, Khan M, Roychoudhury S, Ray K (2002). Distribution of p53 codon 72 polymorphism in Indian primary open angle glaucoma patients. Mol Vis.

[19] Whibley C, Pharoah PDP, Hollstein M (2009). p53 polymorphisms: cancer implications. Nat Rev Cancer.

[20] Cherdyntseva NV, Denisov EV, Litviakov NV, Maksimov VN, Malinovskaya EA, Babyshkina NN (2012). Crosstalk between the FGFR2 and TP53 genes in breast cancer: data from an association study and epistatic interaction analysis. DNA Cell Biol.

[21] Dumont P, Leu JI, Della Pietra AC 3rd, George DL, Murphy M (2003). The codon 72 polymorphic variants of p53 have markedly different apoptotic potential. Nat Genet.

[22] Storey A, Thomas M, Kalita A, Harwood C, Gardiol D, Mantovani F (1998). Role of a p53 polymorphism in the development of human papillomavirus-associated cancer. Nature.

[23] Shi H, Tan SJ, Zhong H, Hu W, Levine A, Xiao CJ (2009). Winter temperature and UV are tightly linked to genetic changes in the p53 tumor suppressor pathway in Eastern Asia. Am J Hum Genet.

[24] Denisov EV, Cherdyntseva NV, Litviakov NV, Malinovskaya EA, Babyshkina NN, Belyavskaya VA, Yue Cheng (2012). TP53 Gene Polymorphisms in Cancer Risk: The Modulating Effect of Ageing, Ethnicity and TP53 Somatic Abnormalities, Tumor Suppressor Genes.

[25] Guo Y, Chen X, Zhang H, Li N, Yang X, Cheng W (2012). Association of OPA1 polymorphisms with NTG and HTG: a meta-analysis. PLoS ONE.

[26] Chen HY, Huang ML, Tsai YY, Hung PT, Lin EJ (2008). Comparing glaucomatous optic neuropathy in primary open angle and primary angle closure glaucoma eyes by scanning laser polarimetry-variable corneal compensation. J Glaucoma.

